# The interplay of DNA methylation over time with Th2 pathway genetic variants on asthma risk and temporal asthma transition

**DOI:** 10.1186/1868-7083-6-8

**Published:** 2014-04-15

**Authors:** Hongmei Zhang, Xin Tong, John W Holloway, Faisal I Rezwan, Gabrielle A Lockett, Veeresh Patil, Meredith Ray, Todd M Everson, Nelís Soto-Ramírez, S Hasan Arshad, Susan Ewart, Wilfried Karmaus

**Affiliations:** 1 Division of Epidemiology, Biostatistics and Environmental Health, School of Public Health, University of Memphis, 236A Robison Hall, Memphis, TN 38152, USA; 2 Department of Epidemiology and Biostatistics, Arnold School of Public Health, University of South Carolina, 800 Sumter Street, Columbia, SC 29208, USA; 3 Clinical and Experimental Sciences, Faculty of Medicine, University of Southampton, University Road, Southampton SO17 1BJ, UK; 4 Human Development and Health, Faculty of Medicine, University of Southampton, University Road, Southampton SO17 1BJ, UK; 5 The David Hide Asthma and Allergy Research Centre, St Mary’s, Hospital, Parkhurst Road, Newport, Isle of Wight PO30 5TG, UK; 6 Department of Large Animal Clinical Sciences, Michigan State University, 3700 East Gull Lake Drive, East Lansing, MI 48824, USA

**Keywords:** Asthma risk, Asthma transition, DNA methylation and SNP interaction, Th2 pathway

## Abstract

**Background:**

Genetic effects on asthma of genes in the T-helper 2 (Th2) pathway may interact with epigenetic factors including DNA methylation. We hypothesized that interactions between genetic variants and methylation in genes in this pathway (*IL4*, *IL4R*, *IL13*, *GATA3*, and *STAT6*) influence asthma risk, that such influences are age-dependent, and that methylation of some CpG sites changes over time in accordance with asthma transition. We tested these hypotheses in subsamples of girls from a population-based birth cohort established on the Isle of Wight, UK, in 1989.

**Results:**

Logistic regression models were applied to test the interaction effect of DNA methylation and SNP on asthma within each of the five genes. Bootstrapping was used to assess the models identified. From 1,361 models fitted at each age of 10 and 18 years, 8 models, including 4 CpGs and 8 SNPs, showed potential associations with asthma risk. Of the 4 CpGs, methylation of cg26937798 (*IL4R*) and cg23943829 (*IL4*) changes between ages 10 and 18 (both higher at 10; *P* = 9.14 × 10^−6^ and 1.07 × 10^−5^, respectively).

At age 10, the odds of asthma tended to decrease as cg12405139 (*GATA3*) methylation increased (log-OR = −12.15; *P* = 0.049); this effect disappeared by age 18. At age 18, methylation of cg09791102 (*IL4R*) was associated with higher risk of asthma among subjects with genotype GG compared to AG (*P* = 0.003), increased cg26937798 methylation among subjects with rs3024685 (*IL4R*) genotype AA (*P* = 0.003) or rs8832 (*IL4R*) genotype GG (*P* = 0.01) was associated with a lower asthma risk; these CpGs had no effect at age 10. Increasing cg26937798 methylation over time possibly reduced the risk of positive asthma transition (asthma-free at age 10 → asthma at age 18; log-OR = −3.11; *P* = 0.069) and increased the likelihood of negative transition (asthma at age 10 → asthma-free at age 18; log-OR = 3.97; *P* = 0.074).

**Conclusions:**

The interaction of DNA methylation and SNPs in Th2 pathway genes is likely to contribute to asthma risk. This effect may vary with age. Methylation of some CpGs changed over time, which may influence asthma transition.

## Background

Asthma is a phenotypically heterogeneous disorder characterized clinically by shortness of breath, wheezing episodes, chest tightness, and acute episodes of coughing [1]. It affects approximately 235 million people worldwide [2], with an estimated 5.4 million cases reported in the UK, including 1.1 million children [3]. While the development of asthma clearly reflects the combination of inherited susceptibility and environmental exposure [4], the disease etiology is poorly understood and biological mechanisms underlying its development are not well established.

Variants in genes encoding T-helper 2 (Th2) cytokines, their receptors, and intracellular signaling pathway components have been consistently associated with asthma in both candidate gene and genome-wide association studies [5-9]. Interleukin (IL)-4 receptor engagement leads to the activation of the transcription factor STAT6, which then upregulates the transcription factor GATA3 [10], which in turn augments the production of the Th2 cytokines IL-4, IL-5, and IL-13 [11]. These gene activities may not only be determined by interactions between transcription factors and cytokine genes, but also affected by epigenetic factors including DNA methylation [12]. Reduction of DNA methylation may facilitate transcription through allowing transcription factors or co-activators to bind to regulatory elements (promoter or enhancer regions) [13-15]. In general, increased DNA methylation of promoter sequences is associated with decreased gene expression [13]. In contrast, intragenic DNA methylation has an inverted U-shape relationship with gene expression levels, whereby the highest levels of intragenic methylation are found in moderately expressed genes [13,16]. However, it is unclear whether the effect of DNA methylation on gene activity is also influenced by SNPs on the same gene as the methylated cytosine-phosphate-guanine (CpG) sites. In addition, there is a possibility that SNPs far away from a CpG site can interact with the CpG site to influence a health outcome, if the SNP is in linkage disequilibrium with another unmeasured SNP close to the CPG site [17].

Genetic variants and DNA methylation play different roles in regulating gene expression and hence disease susceptibility. We have previously shown that interaction between genetic polymorphisms and level of DNA methylation can have “synergistic” effects on risk of disease susceptibility [18-20]. Therefore, we hypothesized that the interaction of genetic variants and DNA methylation in at least some of the genes in the Th2 pathway (*IL4*, *IL4R*, *IL13*, *GATA3*, and *STAT6*) would be associated with the risk of asthma, while SNPs may be a confounder for the main effect of DNA methylation in some genes. We have demonstrated that, during adolescence, some children grow out of asthma, but new asthma diagnoses are also observed [21]. We further hypothesize that association of asthma risk with methylation and with the interaction between DNA methylation and SNP may be significant at one age but absent at another. Finally, we postulate that DNA methylation of some CpG sites changes over time and that temporal changes of DNA methylation are associated with asthma transition over time. We test these hypotheses in subsamples of girls from a population-based birth cohort established on the Isle of Wight (IOW), UK, in 1989 that was aimed to prospectively study the natural history of asthma and allergic conditions.

## Results

Comparing the subsample of 245 female participants with all the 750 female cohort participants, there were no substantial differences (Table 1). For instance, in the IOW cohort, 12% of females had asthma at age 10 and 19% had asthma at age 18. In the subset of 245 females with available methylation data, the corresponding percentages were 11% and 14%, respectively. We also compared other related outcomes including rhinitis, eczema, age of asthma onset, and IgE.

**Table 1:** Characteristics for women with available methylation data compared to the female participants of the total cohort

	**Total female participants n = 750**	**Female with DNA methylation data n = 245**	***P *** **values**
**Factors**	**n (%)**	**n (%)**	
**Asthma at age 18**			
Yes	128 (19.4)	35 (14.3)	0.07
No	531 (80.6)	210 (85.7)	
Missing	91	1	
**Atopy at age 18**			
Yes	159 (35.7)	76 (31.4)	0.29
No	287 (64.3)	166 (68.6)	
Missing	304	3	
**Eczema at age 18**			
Yes	107 (16.3)	37 (15.2)	0.75
No	549 (83.7)	207 (84.8)	
Missing	94	1	
**Rhinitis at age 18**			
Yes	245 (37.2)	86 (35.1)	0.61
No	413 (62.8)	159 (64.9)	
Missing	92	0	
	Mean (STD)	Mean (STD)	
**Age of asthma onset at age 18**	6.95 (5.43)	8.00 (5.41)	0.11
**IgE (log10) at age 18**	2.45 (2.84)	2.35 (2.75)	0.57

We analyzed all available CpG sites in the five genes (*IL4*, *IL4R*, *IL13*, *GATA3*, and *STAT6*) from the Illumina 450 K methylation data and the SNPs that had been genotyped in the cohort for these genes. Summary statistics of these CpG sites and SNPs, including SNP frequencies, mean, and standard deviation of methylation (β) of each CpG site at ages 10 and 18, location of each methylation site, and the corresponding chromosome, are also obtained and included in Additional file 1: Table S1 and Additional file 2: Table S2 and Figures S1 and S2.

### Selected models at ages 10 and 18 years

At each age of 10 and 18 years, up to 1,361 logistic regression models (following exclusion of missing values) were fit to the data. Based on the 34 samples with asthma status and DNA methylation data at age 10 years, this pathway-based selection process did not select any models with a significance level of 0.01. This was as expected, since small samples tend to cause large uncertainty and thus large *P* values. We relaxed the significance level and identified two models showing significance at the level of 0.05. These two models included 2 CpG sites (cg13543854 and cg12405139) and 2 SNPs (rs568727 and rs2229359) in *GATA3* (Tables 2 and 3). Minor allele genotype frequencies lower than 5% were observed for SNPs rs568727 (3.23% for AA in age 10 data) (Additional file 1: Table S1), which is lower than the corresponding population minor allele genotype frequency (~13% from 1,092 human genomes [22]). This further supported the use of bootstrap samples for improving selection accuracy for data with low frequency categorical variables. Utilizing information on asthma status and DNA methylation at age 18 years (n = 245), seven models were identified which either had a significant methylation effect or a significant interaction effect on asthma risk. Three CpG sites and 7 SNPs in 2 genes (*IL4* and *IL4R*) were covered by these seven models (Tables 2 and 3). Two CpG sites (cg09791102 and cg26937798) and 5 SNPs (rs8832, rs1110470, rs1805011, rs1805012, rs3024685) are in *IL4R*, 1 CpG site (cg23943829) and 2 SNPs (rs2070874, rs2243250) are in *IL4*. The distances between the identified CpG sites and the SNPs in each gene are included in Table 4.

**Table 2:** Information on the CpG sites in the selected models before bootstrapping

**Gene**	**CpG sites**	**MapInfo**^**§**^	**Location**	**Chrom.**	**Mean at age 10 (SD)**^**#**^	**Mean at age 18 (SD)**^**#**^	***P *** **value**^**$**^
*GATA3*	cg12405139	8106035	Body	10	0.93 (0.0008)	0.92 (0.011)	0.12
*GATA3*	cg13543854	8095477	TSS200; TSS1500	10	0.047 (0.007)	0.042 (0.008)	5.84 × 10^−3^
*IL4R*	cg09791102	27353414	Body	16	0.94 (0.046)	0.94 (0.050)	0.14
*IL4R*	cg26937798	27326054	5’UTR	16	0.076 (0.022)	0.062 (0.013)	9.14 × 10^−6^
*IL4*	cg23943829	13200911	TSS1500	5	0.88 (0.016)	0.86 (0.019)	1.07 × 10^−5^

^**§**^MapInfo refers to “genomic coordinates on the human genome” and it gives genomic location.

^#^Means and standard deviations (SD) are calculated using beta values. Beta values are used to measure DNA methylation levels of each CpG site.

^$^*P* values are from paired *t*-tests comparing the difference in DNA methylation between ages 10 and 18 years on logit-transformed beta values (to approximate normality).

Mean and SD of DNA methylation of each CpG site are calculated in beta scores.

**Table 3:** Genotype frequencies of the SNPs included in the selected models before bootstrapping based on age 18 SNP data

**Gene**	**SNP**	**Genotype**	n/N (%) at 18 years*	**Gene**	**SNP**	**Genotype**	n/N (%) at 18 years*
*GATA3*	rs568727	AC	95/213 (44.60)	*IL4R*	rs1805012	AG	44/231 (19.05)
		CC	92/213 (43.19)			GG	5/231 (2.16)
		AA	26/213 (12.21)			AA	182/231 (78.79)
*GATA3*	rs2229359	AG	30/234 (12.82)	*IL4R*	rs3024685	AG	104/231 (45.02)
		GG	202/234 (86.32)			GG	42/231 (18.18)
		AA	2/234 (0.85)			AA	85/231 (36.80)
*IL4R*	rs8832	AG	111/233 (47.64)	*IL4*	rs2070874	AG	61/231 (26.41)
		GG	71/233 (30.47)			GG	170/231 (73.59)
		AA	51/233 (21.89)			AA	**—**
*IL4R*	rs1110470	AG	112/227 (49.34)	*IL4*	rs2243250	AG	63/228 (27.63)
		GG	70/227 (30.84)			GG	165/228 (72.37)
		AA	45/227 (19.82)			AA	—
*IL4R*	rs1805011	AC	47/232 (20.26)				
		CC	5/232 (2.15)				
		AA	180/232 (77.59)				

*SNP frequencies at age 10 years are available in Additional file 1.

**Table 4:** Distance of the selected CpG sites to the selected SNPs

**SNP**	**Distance of CpG sites from a SNP (bp)**	
***IL4***	cg23943829	
rs2070874	−599	
rs2243250	−43	
***IL4R***	cg09791102	cg26937798
rs8832	−22,373	−49,733
rs1110470	+16,987	−10,373
rs1805011	−20,458	−47,818
rs1805012	−20,550	−47,910
rs3024685	−23,496	−50,856
***GATA3***	cg13543854	cg12405139
rs568727	−11,224	−666
rs2229359	−5,170	+5,388

+means CpG located after SNP; − CpG located before SNP.

The above identified models were internally assessed using 1,000 bootstrap samples. The selection process introduced earlier was repeated in each of the 1,000 bootstrap samples and the frequency of each model being selected in these bootstrap samples was recorded. Based on the frequencies, we ranked the selected models. For the models examined using age 10 data, the highest frequency was 27 and it corresponded to the model involving cg12405139 and rs2229359. For the model containing cg13543854 and rs568727 identified at age 10, the frequency was 5 out of 1,000 bootstrap samples (Table 5). Due to the small sample size of the age 10 data, low frequencies were expected. However, findings from the model with a frequency of 5 should be interpreted with caution. For the models assessed using age 18 data, the seven identified models discussed earlier were each with a frequency higher than 500 and these frequencies were in the top seven frequencies of all models (Tables 6 and 7). Among the identified CpG sites, according to the SNP annotation file provided by Illumina [23], and further comparison with the SNPs from the 1,092 human genomes (for Caucasians) [22], none of them involve probe SNPs that potentially cause invalid measures of methylation at a CpG site.

**Table 5:** **The effects of SNP, DNA methylation, and their interactions in selected models ( *GATA3 *)**

				**Effects at age 10 (n = 34)**		**Effects at age 18 (n = 233)**	
**Model**	**Model frequency**^**#**^	**Parameter**		**Estimate (log OR)**	***P *** **value**^**$**^	**Estimate (log OR)**	***P *** **value**^**$**^
1	5	cg13543854		−20.27	0.19	3.21	0.066
		rs568727	AA	NA	NA	27.47	0.37
			CC	123.62	**0.04**	−11.50	0.20
			AC	Ref.		Ref.	Ref.
		cg13543854* rs568727	AA	NA	NA	9.42	0.36
			CC	40.76	**0.04**	−3.52	0.21
			AC	Ref.		Ref.	
2	27	cg12405139		−12.15	**0.049**	−0.02	0.99
		rs2229359	AG	−47.21	1.00	−13.86	1.00
			AA	NA	NA	−22.39	0.36
			GG	Ref.		Ref.	Ref.
		cg12405139* rs2229359	AG	12.15	1.00	0.02	1.00
			AA	NA	NA	8.58	0.37
			GG	Ref.		Ref.	Ref.

^#^The frequency is based on 1,000 bootstrap samples.

^$^*P* values significant at the 0.01 level at age 18 are in bold font.

**Table 6:** **The effects of SNP, DNA methylation, and their interactions in selected models ( *IL4R *)**

**Model**	**Model frequency**^#^	**Parameter**		**Effects at age 10 (n = 34); Log-OR (*P *** **value)**	**Effects at age 18 (n = 233); Log-OR (*P *** **value**^**$**^**)**
1	570	cg09791102		8.29 (0.53)	−0.70 (0.2)
		rs1110470	AA	42.92 (0.33)	−3.93 (0.6)
			GG	55.34 (0.24)	−25.00 **(0.004)**
			AG	Ref.	Ref.
		cg09791102* rs1110470	AA	−13.96 (0.34)	1.42 (0.6)
			GG	−18.83 (0.23)	8.51 **(0.003)**
			AG	Ref.	Ref.
2	546	cg09791102		−6.52 (0.18)	3.27 **(0.007)**
		rs1805011	AC	−36.87 (1.00)	16.62 (0.02)
			CC	22.14 (1.00)	−5.22 (1.00)
			AA	Ref.	Ref.
		cg09791102* rs1805011	AC	6.51 (1.00)	−5.88 (0.02)
			CC	NA	−3.27 (1.00)
			AA	Ref.	Ref.
3	571	cg09791102		−6.52 (0.18)	3.34 **(0.006)**
		rs1805012	AG	−36.87 (1.00)	17.16 (0.02)
			GG	22.14 (1.00)	−5.01 (1.00)
			AA	Ref.	Ref.
		cg09791102* rs1805012	AG	6.52 (1.00)	−6.03 (0.02)
			GG	NA	−3.34 (1.00)
			AA	Ref.	Ref.
4	633	cg26937798		−4.91 (0.23)	1.53 (0.3)
		rs3024685	AG	22.65 (0.11)	−24.15 **(0.002)**
			GG	−4.59 (1.00)	0.62 (0.9)
			AA	Ref.	Ref.
		cg26937798* rs3024685	AG	8.63 (0.11)	−8.37 **(0.003)**
			GG	4.91 (1.00)	0.09 (0.9)
			AA	Ref.	Ref.
5	761	cg26937798		0.01 (1.00)	0.09 (0.9)
		rs8832	AA	−16.93 (1.00)	10.38 (0.07)
			GG	−29.34 (0.59)	−20.81 **(0.01)**
			AG	Ref.	Ref.
		cg26937798* rs8832	AA	−0.01 (1.00)	3.73 (0.09)
			GG	−11.56 (0.59)	−7.23 **(0.01)**
			AG		Ref.

^#^The frequency is based on 1,000 bootstrap samples.

^$^*P* values significant at the 0.01 level at age 18 are in bold font.

**Table 7:** **The effects of SNP, DNA methylation, and their interaction effects in selected models ( *IL4 *)**

**Model**	**Model frequency**^#^	**Parameter**		**Effects at age 10 (n = 34); Log-OR (*P *** **value)**	**Effects at age 18 (n = 233); Log-OR (*P *** **value)**^**$**^
6	532	cg23943829		−2.17 (1.00)	−3.60 **(0.009)**
		rs2070874	AG	−22.16 (1.00)	−4.97 (0.4)
			GG	Ref.	Ref.
		cg23943829* rs2070874	AG	2.17 (1.00)	2.61 (0.4)
			GG	Ref.	Ref.
7	669	cg23943829		−1.60 (0.64)	−4.47 **(0.003)**
		rs2243250	AG	−21.18 (1.00)	−6.75 (0.2)
			GG	Ref.	Ref.
		cg23943829* rs2243250	AG	1.60 (1.00)	3.84 (0.1)
			GG	Ref.	Ref.

^#^The frequency is based on 1,000 bootstrap samples.

^$^*P* values significant at the 0.01 level at age 18 are in bold font.

### DNA methylation and odds of asthma at ages 10 and 18

At age 10 years, the selected model showed a tendency that the odds of asthma decreased as the DNA methylation of cg12405139 increased (log-OR = −12.15 with *P* = 0.049). The effect of DNA methylation at this CpG site disappeared at age 18 (Table 5). For CpG site cg13543854, based on age 10 data, subjects with genotype CC with an increase of DNA methylation also had an increased odds of asthma compared to subjects with genotype AC (*P* for the interaction effect = 0.04). However, due to the low frequency in which its corresponding model was selected in 1,000 bootstrap samples, this finding should be interpreted with caution. At age 18, of the CpG sites and SNPs in the seven selected models, some were potential risk factors and others were possibly protective (Tables 6 and 7). For *IL4R*, methylation at cg09791102 was a risk factor (models 2 and 3 in Table 6, log-OR = 3.27 with *P* = 0.007, and log-OR = 3.34 with *P* = 0.006). The interaction of methylation at site cg09791102 with the rs1110470 genotype GG (model 1, *P* = 0.003) suggested that the DNA methylation of this site was associated with higher risk of asthma among girls with genotypes GG compared to girls with genotype AG (Table 6). Within *IL4R*, the interaction of cg26937798 with rs3024685 and with rs8832 (models 4 and 5, *P* = 0.003 and 0.01, respectively) indicated that increased DNA methylation of this site among subjects with the rs3024685 genotype AA or rs8832 genotype GG was associated with a lower risk of asthma. It is worth noting that none of the CpG sites showing a significant interaction with SNPs is close to those SNPs (Table 5). We also observed that rs1805011 and rs1805012 in *IL4R* were potential confounders in that they do not statistically significantly interact with CpG site cg09791102, however, cg09791102 became an insignificant factor (*P* = 0.51) if these two SNPs were excluded (data not shown). For *IL4*, DNA methylation of cg23943829 was protective (models 6 and 7 in Table 7; log-OR = −3.60 with *P* = 0.009, and log-OR = −4.47 with *P* = 0.003, respectively). These CpG sites identified in the age 18 data were also tested within the age 10 data, but did not show any significant main or interaction effects (Tables 6 and 7).

### DNA methylation change and asthma transition

Before presenting the findings of the association of DNA methylation changes with asthma status transitions, we focus on the 4 CpG sites included in the selected models and first discuss the stability of DNA methylation at these CpG sites (cg12405139 [*GATA3*], cg09791102 [*IL4R*], cg26937798 [*IL4R*], and cg23943829 [*IL4*]) between ages 10 and 18. The level of DNA methylation at each of these 4 CpG sites is consistent between the two ages in that, if a site was highly methylated at age 10, then it was also highly methylated at age 18. Further results from paired *t*-tests indicated that methylation of two CpG sites, cg12405139 and cg09791102, does not show any statistically significant changes between the two ages (Table 2), while methylation of CpG sites cg13543854, cg26937798, and cg23943829 at age 10 is statistically significantly higher than the methylation of these two sites at age 18 (means of pair differences in the logit scale are 0.14, 0.24 and 0.17 with *P* = 5.84 × 10^–3^, 9.14 × 10^−6^, and 1.07 × 10^−5^, respectively; Table 2).

For the CpG sites where methylation is stable between ages 10 and 18 years, we evaluated the association of asthma transition with DNA methylation at age 18, SNP, and their interaction. No significant main or interaction effects were identified (Table 8). For the 2 CpG sites where methylation changed between ages 10 and 18 years (cg23943829 and cg26937798), we evaluated the association of asthma transition with change of DNA methylation between 10 and 18 years, SNP, and their interaction. Although we did not detect a statistically significant interaction effect, the increase of DNA methylation of cg26937798 over time had a tendency to reduce the risk of positive transition (log-OR = −3.11; *P* = 0.069) and increased the likelihood of negative transition (log-OR = 3.97; *P* = 0.074, Table 8).

**Table 8:** The effects of changes in DNA methylation on asthma transition between ages 10 and 18

**Gene**	**CpG sites**	**Effects on transition; Log-OR (*P *** **value)**	
		**(−,+)**^**#**^	**(+,–)**^**$**^
Dynamic methylation			
*GATA3*	cg13543854	1.69 (0.22)	−0.58 (0.78)
*IL4R*	cg26937798	−3.11 (0.069)	3.97 (0.074)
*IL4*	cg23943829	3.56 (0.11)	−0.99 (0.73)
Stable methylation			
*GATA3*	cg12405139	−1.60 (0.64)	1.06 (0.71)
*IL4R*	cg09791102	−21.18 (1.00)	−0.67 (0.47)

^#^(−,+): positive transition of asthma between ages 10 and 18 years.

^$^(+,–): negative transition of asthma between ages 10 and 18 years.

## Discussion

As previously demonstrated, interactions between DNA methylation and SNPs may show a stronger effect compared to the effects of SNPs alone on the risk of asthma [18,19]. Utilizing a comprehensive assessment of the association of asthma with methylated CpG sites, SNPs, and their interactions in genes within the Th2 pathway, we identified CpG sites such that their main effects or interaction effects with SNPs were potentially associated with the risk of asthma. We also explored whether the changes of methylation between ages 10 and 18 is associated with the asthma transition. Based on our knowledge, this is the first study to systematically assess the DNA methylation effects and their interaction with SNPs in the Th2 pathway on asthma risk and, more importantly, the first study testing the effect of temporal change of DNA methylation on asthma transition between ages 10 and 18.

We examined CpG sites and SNPs in 5 genes (*IL4*, *IL4R*, *IL13*, *GATA3*, and *STAT6*) related to Th2 immunity. Testing the effects of genetic and epigenetic variants targeted at a specific asthma-related pathway has the potential to gain a higher statistical power compared to the unbiased approach of investigating the whole genome. The CpG sites and SNPs were selected based on a consideration of main methylation effect and the interactions between methylation and SNPs. In total, seven models based on age 18 data were identified. For the age 10 data, we did not identify any statistically significant models based on significance level 0.01, but did detect two models possibly of interest at significance level 0.05. Through further internal validation using 1,000 bootstrapping samples, one of the two models identified at age 10 was with low selection frequency while all the seven identified models from the age 18 data were with a frequency higher than 500 out of 1,000. In total, these models included 5 CpG sites and 9 SNPs. An alternative approach to select models is a direct utilization of bootstrap samples: first using bootstrap samples to improve the estimates of standard errors of effect estimates, and then identifying models based on improved test statistics [24].

We found that the methylation of individual CpG sites provided limited evidence of associations with asthma status. In contrast, the interaction effects between SNPs and DNA methylation of those CpG sites were statistically stronger. For instance, cg09791102 was an insignificant factor by itself (*P* = 0.51). However, this CpG site showed a significant interaction effect with rs1110470 and became a potential risk factor for girls with genotypes GG compared to AG (model 1, *P* = 0.003; Table 6). All the CpG sites showing a significant interaction with SNPs were not close to the SNPs, indicating possible biological independence between those CpG sites and SNPs. A recent study indicated that such observation might also be due to these SNPs being in linkage disequilibrium with SNPs close to those CpG sites [17]. We further noticed that rs1805011 and rs1805012 in *IL4R* were potential confounders in that they do not statistically significantly interact with CpG site cg09791102; however, cg09791102 became an insignificant factor (*P* = 0.51) if these two SNPs were excluded.

The model identified using age 10 DNA methylation data and asthma status information indicated a substantial decrease in the odds of asthma along with the increase of DNA methylation of cg12405139 (log-OR = −12.15 with *P* = 0.049). Although its corresponding *P* value was not significant at the significance level of 0.01, the statistical significance was completely lost (*P* = 0.99) when evaluating age 18 data. In contrast, the seven selected models based on age 18 data were not significant to data collected at age 10. It is possible that this lack of association at age 10 was due to small sample size (n = 34). On the other hand, the results from paired *t*-tests showed a significant change in methylation for two methylation sites (cg23943829 and cg26937798) between ages 10 and 18. This indicates that the significant effects of these two sites or the effects of their interaction with SNP on asthma status at age 18 were likely to be due to change of methylation over time. A possible explanation is that methylation levels at some CpG sites were dynamic during the period of adolescent transition and were only associated with disease status at one time point. We also observed that at some CpG sites the effects of DNA methylation or its interaction with SNPs were attenuated at age 18 (Tables 6 and 7). Since the sample size at age 10 is much smaller than that at age 18, a larger variation in the age 10 data was expected. A further investigation to assess the attenuation is needed in larger samples. Furthermore, although we did not detect any significant interaction effects between methylation change (from ages 10 to 18) for cg26937798 and its corresponding SNPs, the tendency of methylation increase of cg26937798 over time reducing the risk of positive transition and increasing the likelihood of asthma remission is informative and certainly deserves further investigation.

The selection of CpG sites and SNPs was based on a model selection approach with each model composed of one CpG site and one SNP. To further assess the findings, we used 1,000 bootstrap samples to internally validate the identified CpG sites. The bootstrapping technique has been studied and used in various studies for the purpose of internal validation and shown to be effective [25]. The exclusion of the model involving cg13543854 and rs568727 due to a low frequency of selection among the 1,000 bootstrap samples indicates that this model, identified based on the original data, is likely to be a random finding. Overall, the findings from this work provided insight into the understanding of the impact of methylation on asthma at a single time point (ages 10 or 18) and on asthma transition between ages 10 and 18.

We did not perform technical replicates on DNA methylation analyses. However, various studies have shown its overall congruence with quantitative pyrosequencing [26], validated its robustness via cancer cell lines [27], and confirmed the quality of pre-processed methylation data [28]. Another potential limitation exists in the influence of cell composition on the measure of DNA methylation. DNA methylation obtained from peripheral blood leukocytes served as surrogate measures of cell mixture distribution [29]. These surrogate measures might introduce noise in the association studies presented in our work and consequently cause power loss in our inferences. However, the estimates are still expected to be unbiased as seen in the modeling proposed earlier [29]. In the logistic regression for identifying CpG sites and SNPs potentially associated with asthma, each model included one CpG site and one SNP in their corresponding gene. Since SNP × SNP and CpG × CpG interactions may also exist, there is a possibility that our selection might have overlooked some potentially important CpG sites or SNPs due to interaction effects.

Multiple testing was not corrected using the commonly used methods such as the method controlling for false discovery rate. We, instead, used the methods built upon bootstrapping to further assess the selected models as in previous studies. It has been shown, and also seen in our recent simulation studies, that the false discovery rate-based multiple testing correction method cannot control for type I error [30,31]. However, this type of concern will be minimal when the sample size is large. In addition, this study only included girls. Gender difference in DNA methylation has been noted in various studies [32-35]. It is possible that the identified CpG sites associated with asthma or its transition are different if analyzed in boys. Finally, since this is the first study that examined the methylation change during adolescent transition and its association with asthma status transition, a further validation study in a comparable population and a thorough investigation of all the selected CpG sites and SNPs along with other adjusting factors are needed. These further investigations need careful epidemiological and biological justification, and are our on-going work.

## Conclusions

The interaction of DNA methylation and SNPs in Th2 pathway genes contribute to the risk of asthma, but effects may not be stable over time. DNA methylation of some CpG sites changes over time and DNA methylation change has the potential to influence asthma transition.

## Methods

### Data collection

The study cohort was composed of children born between January 1, 1989 and February 28, 1990 on the IOW, UK, a semi-rural island without heavy industry near the UK mainland [36]. Of the 1,536 children born and recruited in this period, 1,456 were available for further follow-up. In follow-ups, data were collected at ages 1, 2, 4, 10, and 18 years. Questionnaires that included the questions of the International Study of Asthma and Allergy in Childhood (ISAAC) [37] were administered at each follow-up. In the case that parents or participants could not attend a follow-up visit to complete the questionnaire, a modified version was given either over the telephone or via mail. Information such as disease status (e.g., asthma, eczema, and rhinitis), age of asthma onset, puberty events, and smoking status were collected based on responses to the questionnaires.

Peripheral blood samples for DNA isolation were collected at ages 10 and 18 years. At age 18 years, saliva samples were also collected. Genotyping was conducted on samples from 1,211 cohort subjects. DNA methylation of CpG sites was determined in DNA extracted from peripheral blood leucocytes collected at 18 years of age from 245 female cohort participants. These 245 participants were randomly selected from the 750 female cohort participants for screening of epigenetic associations with allergic disorders in a mother-infant study. Random sampling was used to reduce bias in the subsample. Of these 245 women, we randomly sampled 16 individuals with asthma and 18 individuals without, and obtained their DNA methylation at age 10 measured from peripheral blood leucocytes.

The outcome considered was occurrence of asthma at ages 10 and 18 based on the questions of ISAAC [37]. It was defined as having “ever had asthma” and either “wheezing or whistling in the chest in the last 12 months” or “current treatment for asthma”. Wheezing was defined as wheezing or whistling in the last 12 months.

### Transition of asthma status

We consider two types of asthma transitions, positive and negative, from ages 10 to 18. Asthma status was treated as a binary variable (0/1), so at each age a participant was classified either as asthma-free (0) or as having asthma (1). A subject experienced positive transition of asthma if he/she was asthma-free at age 10 but had asthma at age 18. Negative transition (also called remission) occurred when an asthma patient outgrew the disease at age 18.

### The SNPs and CpG sites in the five genes

We selected SNPs potentially related to asthma by use of a tagging strategy for each of the five genes in the Th2 pathway (*IL4*, *IL4R*, *IL13*, *GATA3*, and *STAT6*). In this tagging strategy, SNPs that had previously been associated with asthma and/or were potentially functional variants were prioritized. Specifically, Tagger implemented in Haploview using HapMap Caucasian data was used to develop the tagging scheme across each gene including 10 kb upstream and downstream of each gene with *r*^*2*^ of 0.85 as the tagging threshold. Genotyping was conducted on DNA extracted from blood or saliva samples for 1,211 cohort subjects using GoldenGate Genotyping Assays (Illumina, Inc., CA, USA). Details of genotyping are reported elsewhere [18].

For methylation analyses, DNA was extracted from whole blood using a standard salting out procedure [38]. DNA concentration was determined by PicoGreen dsDNA quantitation kit (Molecular Probes, Inc., OR, USA). One microgram of DNA was bisulfite-treated for cytosine to thymine conversion using the EZ 96-DNA methylation kit (Zymo Research, CA, USA), following the manufacturer’s standard protocol. Genome-wide DNA methylation was assessed using the Illumina Infinium HumanMethylation450 BeadChip (Illumina, Inc., CA, USA), which interrogates >484,000 CpG sites associated with approximately 24,000 genes. Arrays were processed using a standard protocol as described elsewhere [39], with multiple identical control samples assigned to each bisulfite conversion batch to assess assay variability and samples randomly distributed on microarrays to control against batch effects. The BeadChips were scanned using a BeadStation, and the Methylation Module of GenomeStudio (Version 2011.1) software calculated the methylation level for each queried CpG as beta (*β*) values. They represent the proportions of intensity of methylated (*M*) over the sum of methylated and unmethylated (*U*) sites (*β = M/[c + M + U*] with constant c introduced for the situation of too small *M + U*). The methylation data were then preprocessed using the Bioconductor IMA package and the ComBat function in R for initial quality control to remove unreliable CpG sites, correct for probe types, remove background noise, and correct for batch effect [40,41]. DNA methylation of 100 CpG sites and 42 SNPs were available for the genes *IL4*, *IL4R*, *IL13*, *GATA3*, and *STAT6* and included in the analyses. Specifically, 5 CpG sites and 4 SNPs for *IL4*, 10 CpG sites and 13 SNPs for *IL4R*, 9 CpG sites and 7 SNPs for *IL13*, 67 CpG sites and 17 SNPs for *GATA3*, and 9 CpG sites and 1 SNP for *STAT6* were epityped and genotyped.

### Statistical analysis

To assess whether the analytical sample (245 female participants) was representative of the female cohort available at age 18 years, we applied two types of tests. The χ^2^ tests were used to assess the agreement between the subsample (n = 245) and the female cohort on the prevalence of asthma, atopy, eczema, and rhinitis. Two sample *t*-tests were used to assess the agreement on IgE and ages of asthma onset.

To identify statistically significant interaction effects on asthma among the candidate CpG sites and SNPs on these five genes, logistic regression models were applied. Asthma status was the dependent variable and SNPs, DNA methylation in logit transformed *β* values, and their interaction were the independent variables. For each gene, we considered models formed by all possible combinations between SNPs and methylation sites. For instance, 67 CpG sites and 17 SNPs were available for *GATA3* and thus 1,139 (67 CpG sites × 17 SNPs) logistic regression models were fitted for *GATA3*. Models that showed either statistically significant main effects of DNA methylation or significant interaction effects of DNA methylation and SNPs were chosen as candidate models.

To evaluate methylation changes between ages 10 and 18 on the selected CpG sites, paired *t*-tests were applied to logit-transformed methylation values. To evaluate whether DNA methylation change at the selected CpG sites was associated with the odds of asthma transition (positive and negative) with respect to no asthma at both ages while taking SNP effects into account, logistic regressions were applied to test the effects of methylation change, SNP, and their interaction. For CpG sites where DNA methylation did not change over time, we evaluated whether DNA methylation or its interaction with SNPs was associated with the odds of asthma transition (positive and negative) compared to no asthma at both ages. In this direction, logistic regressions were also used and DNA methylation at age 18 of the selected CpG sites along with corresponding SNPs were included in the analyses.

Since the selection of the genes was guided by biological pathways, the CpG sites were not independent. To take the multiple testing into account while admitting the dependence between tests in this selection process, we set the significance level at a lower level, specifically, 0.01, aiming to identify models with possibly significant CpG sites and SNPs. To further assess the identified models, bootstrapping was then used to evaluate the identified models at each age. Each bootstrap sample was created based on random sampling by drawing with replacement and the size of each bootstrap sample is the same as in the original sample. In total, 1,000 bootstrapping samples were used, logistic regression models with all possible combination of CpG sites and SNPs within a gene were fitted to each sample, and the relative frequency of each selected model out of 1,000 bootstrapping samples was recorded. The bootstrapping technique used for variable and model selection was proposed in the 1990’s [42] and has been discussed and applied in different studies [43,44]. In our study, since minor allele frequencies for some SNPs were low, using bootstrap samples had the potential to reduce the bias on variable selections due to large variations in the estimation of variances caused by low minor allele frequencies. We note that models with CpG sites involving probe SNPs would not be considered due to biased measures of DNA methylation. The significance level of 0.05 was used for the remaining tests with respect to the identified CpG sites and SNPs; these tests include *t*-tests and logistic regressions. All the statistical analyses were performed using the R statistical computing package [45].

## Abbreviations

CpG: Cytosine-phosphate-guanine; IL: Interleukin; IOW: Isle of Wight; ISAAC: International Study of Asthma and Allergy in Childhood; OR: Odds ration; Th2: T-helper 2.

## Competing interests

The authors declare that they have no competing interests.

## Authors’ contributions

HZ conducted the statistical analysis, interpreted the data, and drafted the manuscript. XT, TME, MR, and NSR contributed to statistical analysis. JWH supervised the assessment of DNA methylation. FIR and GAL contributed to the assessment of DNA methylation. SE selected and measured the single nucleotide polymorphisms. HZ, SE, WK, and SHA contributed to funding acquisition. HZ, WK, JWH, SHA, and SE participated in the design of the study. SHA and VP supervised clinical assessment of the cohort children. All authors participated in the revision of the manuscript, and all authors read and approved the final manuscript.

## Supplementary Material

Additional file 1: Table S1 Genotype frequencies of the 42 SNPs at ages 10 and 18 years. Click here for file

Additional file 2: Table S2 Information on the candidate CpG sites in the Th2 pathway. Mean and SD are calculated in beta values and for ages 10 and 18 years. **Figure S1**. The mean and SD of CpG sites in genes *IL4*, *IL4R*, *IL13*, and *STAT6* (data are in Additional file 2: Table S2.). **Figure S2.** The mean and SD of CpG sites in the *GATA3* gene (data are in Additional file 2: Table S2). Click here for file
