# Supplementary material for: The interplay of DNA methylation over time with Th2 pathway genetic variants on asthma risk and temporal asthma transition
Source: Clin Epigenetics. 2014 Apr 15;6(1):8. doi: 10.1186/1868-7083-6-8 (PMC4023182; doi:10.1186/1868-7083-6-8)
Supplement: Additional file 1: Table S1 — Genotype frequencies of the 42 SNPs at ages 10 and 18 years. [file 1868-7083-6-8-S1.docx]

**Appendix**

Table A1. Genotype frequencies of the 42 SNPs at ages 10 and 18 years.

| **Gene** | **SNP** | **Genotype** | **n/N(%)[age10]** | **n/N(%)[age18]** | ***Gene*** | **SNP** | **Genotype** | **n/N(%)[age10]** | **n/N(%)[age18]** |
| --- | --- | --- | --- | --- | --- | --- | --- | --- | --- |
| *IL4* | rs2070874 | AG | 9/34 (26.47) | 61/231 (26.41) | *IL4* | rs2227284 | AC | 12/33 (36.36) | 93/234 (39.74) |
|  |  | GG | 25/34 (73.53) | 170/231 (73.59) |  |  | CC | 19/33 (57.58) | 131/234(55.98) |
|  |  | AA | **--** | **--** |  |  | AA | 2/33 (6.06) | 10/234 (4.27) |
| *IL4* | rs2243250 | AG | 10/32 (31.25) | 63/228 (27.63) | *IL4* | rs2243263 | CG | 4/33 (12.12) | 39/233 (16.74) |
|  |  | GG | 22/32 (68.75) | 165/228(72.37) |  |  | GG | 1/33 (3.03) | 3/233 (1.29) |
|  |  | AA | -- | -- |  |  | CC | 28/33 (84.85) | 191/233 (81.97) |
| *IL4R* | rs8832 | AG | 19/33 (57.58) | 111/233 (47.64) | *IL4R* | rs1110470 | AG | 12/32 (37.5) | 112/227 (49.34) |
|  |  | GG | 8/33 (24.24) | 71/233 (30.47) |  |  | GG | 11/32 (34.38) | 70/227 (30.84) |
|  |  | AA | 6/33 (18.18) | 51/233 (21.89) |  |  | AA | 9/32 (28.13) | 45/227 (19.82) |
| *IL4R* | rs1805011 | AC | 9/33 (27.27) | 47/232 (20.26) | *IL4R* | rs1805012 | AG | 9/33 (27.27) | 44/231 (19.05) |
|  |  | CC | 1/33 (3.03) | 5/232 (2.15) |  |  | GG | 1/33 (3.03) | 5/231 (2.16) |
|  |  | AA | 23/33 (69.70) | 180/232 (77.59) |  |  | AA | 23/33 (69.70) | 182/231 (78.79) |
| *IL4R* | rs2057768 | AG | 17/34 (50.00) | 106/234 (45.3) | *IL4R* | rs3024604 | AG | 5/33 (15.15) | 34/230 (14.78) |
|  |  | GG | 14/34 (41.18) | 112/234 (47.86) |  |  | GG | -- | 1/230 (0.43) |
|  |  | AA | 3/34 (8.82) | 16/234 (6.84) |  |  | AA | 28/33 (84.85) | 195/230 (84.78) |
| *IL4R* | rs3024622 | CG | 16/30 (53.33) | 104/241 (47.06) | *IL4R* | rs3024676 | AC | 13/33 (39.39) | 65/232 (28.02) |
|  |  | GG | 11/30 (36.67) | 91/241 (41.18) |  |  | CC | 19/33 (57.78) | 161/232(69.40) |
|  |  | CC | 3/30 (10.00) | 26/241 (11.76) |  |  | AA | 1/33 (3.03) | 6/232 (2.59) |
| *IL4R* | rs3024685 | AG | 13/34 (38.24) | 104/231 (45.02) | *IL4R* | rs4787423 | AG | 10/34 (29.41) | 53/234 (22.65) |
|  |  | GG | 7/34 (20.59) | 42/231 (18.18) |  |  | GG | -- | 3/234 (1.28) |
|  |  | AA | 14/34 (41.18) | 85/231 (36.80) |  |  | AA | 24/34 (70.59) | 178/234(76.07) |
| Table A1. Genotype frequencies of the 42 SNPs at ages 10 and 18 years (continued). | | | | | | | | | |
| **Gene** | **SNP** | **Genotype** | **n/N(%)[age10]** | **n/N(%)[age18]** | ***Gene*** | **SNP** | **Genotype** | **n/N(%)[age10]** | **n/N(%)[age18]** |
| *IL4R* | rs6498012 | CG | 11/33 (33.33) | 120/234 (51.28) | *IL4R* | rs12102586 | AG | 7/34 (20.59) | 43/234 (18.38) |
|  |  | GG | 15/33 (45.45) | 77/234 (32.91) |  |  | GG | 27/34 (79.41) | 189/234(80.77) |
|  |  | CC | 7/33 (21.21) | 37/234 (15.81) |  |  | AA | -- | 2/234 (0.85) |
| *IL4R* | rs16976728 | AG | 17/33 (51.52) | 99/232 (42.67) | *IL13* | rs20541 | AG | 8/34 (23.53) | 66/235 (28.09) |
|  |  | GG | 12/33 (36.36) | 88/232 (37.93) |  |  | GG | 24/34 (70.59) | 159/235 (67.66) |
|  |  | AA | 4/33 (12.12) | 45/232 (19.40) |  |  | AA | 2/34 (5.88) | 10/235 (4.26) |
| *IL13* | rs1295683 | AG | 6/34 (17.65) | 41/233 (17.60) | *IL13* | rs1295685 | AG | 8/34 (23.53) | 67/234 (28.63) |
|  |  | GG | 28/34 (82.35) | 189/233 (81.12) |  |  | GG | 24/34 (70.59) | 157/234(67.09) |
|  |  | AA | -- | 3/233 (1.29) |  |  | AA | 2/34 (5.88) | 10/234 (4.27) |
| *IL13* | rs1800925 | AG | 12/34 (35.29) | 67/233 (28.76) | *IL13* | rs1881457 | AC | 12/33 (36.36) | 66/232 (28.45) |
|  |  | GG | 22/34 (64.71) | 158/233 (67.81) |  |  | AA | 21/33 (63.64) | 159/232 (68.53) |
|  |  | AA | -- | 8/233 (3.43) |  |  | CC | -- | 7/232 (3.02) |
| *IL13* | rs2069743 | AG | -- | 1/236 (0.42) | *IL13* | rs2243204 | AG | 5/33 (15.15) | 37/235 (15.74) |
|  |  | GG | -- | -- |  |  | GG | 28/33 (84.85) | 198/235(84.26) |
|  |  | AA | 33/33 (100.0) | 235/236 (99.58) |  |  | AA | -- | -- |
| *GATA3* | rs369421 | AG | 16/34 (47.06) | 71/235 (30.21) | *GATA3* | rs406103 | AG | 8/33 (24.24) | 78/233 (33.48) |
|  |  | GG | -- | 5/235 (2.13) |  |  | GG | 24/33 (72.73) | 142/233 (60.94) |
|  |  | AA | 18/34 (52.94) | 159/235 (67.66) |  |  | AA | 1/33 (3.03) | 13/233 (5.58) |
| *GATA3* | rs422628 | AG | 14/33 (42.42) | 93/233 (39.91) | *GATA3* | rs434645 | AG | 10/33 (30.3) | 67/229 (29.26) |
|  |  | GG | 2/33 (6.06) | 12/233 (5.15) |  |  | GG | 23/33 (69.7) | 158/229 (69.00) |
|  |  | AA | 17/33 (51.52) | 128/233 (54.94) |  |  | AA | -- | 4/229 (1.75) |
| *GATA3* | rs477461 | AG | 6/33 (18.18) | 63/233 (27.04) | *GATA3* | rs568727 | AC | 18/31 (58.06) | 95/213 (44.60) |
|  |  | GG | -- | 5/233 (2.15) |  |  | CC | 12/31 (38.71) | 92/213 (43.19) |
| Table A1. | Genotype frequencies of the 42 SNPs at ages 10 and 18 years (continued). | | | | | | | |  |
| **Gene** | **SNP** | **Genotype** | **n/N(%)[age10]** | **n/N(%)[age18]** | ***Gene*** | **SNP** | **Genotype** | **n/N(%)[age10]** | **n/N(%)[age18]** |
|  |  | AA | 27/33 (81.82) | 165/233 (70.82) |  |  | AA | 1/31 (3.23) | 26/213 (12.21) |
| *GATA3* | rs574495 | AG | 19/33 (57.78) | 123/233 (52.79) | *GATA3* | rs1058240 | AG | 11/33 (33.33) | 73/232 (31.47) |
|  |  | GG | 8/33 (24.24) | 63/233 (27.04) |  |  | GG | -- | 8/232 (3.45) |
|  |  | AA | 6/33 (18.18) | 47/233 (20.17) |  |  | AA | 22/33 (66.67) | 151/232 (65.09) |
| *GATA3* | rs10905277 | AG | 17/32 (53.12) | 107/225 (47.56) | *GATA3* | rs12412241 | AG | 17/34 (50.00) | 92/232 (39.66) |
|  |  | GG | 8/32 (25.00) | 57/225 (25.33) |  |  | GG | 15/34 (44.12) | 121/232 (52.16) |
|  |  | AA | 7/32 (21.88) | 61/225 (27.11) |  |  | AA | 2/34 (5.88) | 19/232 (8.19) |
| *GATA3* | rs1269486 | AG | 18/34 (52.94) | 87/230 (37.83) | *GATA3* | rs2229359 | AG | 3/34 (8.82) | 30/234 (12.82) |
|  |  | GG | 13/34 (38.24) | 130/230 (56.52) |  |  | GG | 31/34 (91.18) | 202/234 (86.32) |
|  |  | AA | 3/34 (8.82) | 13/230 (5.65) |  |  | AA | -- | 2/234 (0.85) |
| *GATA3* | rs2229360 | AG | -- | 3/236 (1.27) | *GATA3* | rs3802600 | AT | 8/34 (23.53) | 73/231 (31.6) |
|  |  | GG | 34/34 (100.0) | 233/236 (98.73) |  |  | TT | 26/34 (76.47) | 149/231 (64.5) |
|  |  | AA | -- |  |  |  | AA | -- | 9/231 (3.9) |
| *GATA3* | rs3802604 | AG | 16/32 (50.00) | 103/229 (44.98) | *GATA3* | rs3824662 | AC | 8/34 (23.53) | 69/233 (29.61) |
|  |  | GG | 3/32 (9.38) | 38/229 (16.59) |  |  | CC | 26/34 (76.47) | 156/233 (66.95) |
|  |  | AA | 13/32 (40.62) | 88/229 (38.43) |  |  | AA | -- | 8/233 (3.43) |
| *GATA3* | rs10752126 | CG | 20/32 (62.50) | 102/231 (44.16) | *STAT6* | rs1059513 | AG | 5/33 (15.15) | 45/233 (19.31) |
|  |  | GG | 3/32 (9.38) | 49/231 (21.21) |  |  | GG | -- | 2/233 (0.86) |
|  |  | CC | 9/32 (28.12) | 80/231 (34.63) |  |  | AA | 28/33 (84.85) | 186/233 (79.83) |
