# Supplementary material for: The interplay of DNA methylation over time with Th2 pathway genetic variants on asthma risk and temporal asthma transition
Source: Clin Epigenetics. 2014 Apr 15;6(1):8. doi: 10.1186/1868-7083-6-8 (PMC4023182; doi:10.1186/1868-7083-6-8)

Table A2. Information on the candidate CpG sites in the Th2 pathway. Mean and SD are calculated in beta values and for ages 10 and 18 years.

| **Gene** | **Name** | **Mean (SD) age10** | **Mean (SD) age18** | **MapInfo^§^** | **Location** | **Chromosome_36** |
| --- | --- | --- | --- | --- | --- | --- |
| *IL4* | cg12377972 | 0.910 (0.026) | -- | 132037529 | 5'UTR;1stExon | 5 |
| *IL4* | cg18145937 | 0.905 (0.014) | 0.911 (0.014) | 132038639 | Body | 5 |
| *IL4* | cg23943829 | 0.879 (0.016) | 0.864 (0.019) | 132037010 | TSS1500 | 5 |
| *IL4* | cg25368824 | 0.872 (0.025) | -- | 132037251 | TSS200 | 5 |
| *IL4* | cg26787239 | 0.901 (0.017) | -- | 132036424 | TSS1500 | 5 |
| *IL4R* | cg01165142 | 0.747 (0.019) | 0.619 (0.044) | 27367172 | Body | 16 |
| *IL4R* | cg01706029 | 0.050 (0.005) | 0.045 (0.005) | 27325672 | 5'UTR | 16 |
| *IL4R* | cg05729093 | 0.036 (0.006) | 0.036 (0.008) | 27324953 | TSS1500 | 16 |
| *IL4R* | cg05903710 | 0.946 (0.007) | 0.936 (0.011) | 27375732 | 3'UTR | 16 |
| *IL4R* | cg08317580 | 0.956 (0.009) | 0.957 (0.012) | 27345891 | 5'UTR | 16 |
| *IL4R* | cg08932316 | 0.945 (0.007) | 0.953 (0.009) | 27324341 | TSS1500 | 16 |
| *IL4R* | cg09791102 | 0.944 (0.046) | 0.942 (0.05) | 27353414 | Body | 16 |
| *IL4R* | cg16649560 | 0.214 (0.033) | 0.178 (0.034) | 27338391 | 5'UTR | 16 |
| *IL4R* | cg26937798 | 0.076 (0.022) | 0.062 (0.013) | 27326054 | 5'UTR | 16 |
| *IL4R* | cg06641959 | -- | 0.087 (0.019) | 27325254 | 5'UTR;1stExon | 16 |
| *IL13* | cg04303330 | 0.347 (0.041) | -- | 132020329 | TSS1500 | 5 |
| *IL13* | cg06584121 | 0.897 (0.014) | 0.867 (0.026) | 132021717 | TSS200 | 5 |
| Table A2. Information on the candidate CpG sites in the Th2 pathway. Mean and SD are calculated in beta values and for ages 10 and 18 years (continued) | | | | | | |
| **Gene** | **Name** | **Mean (SD) age10** | **Mean (SD) age18** | **MapInfo^§^** | **Location** | **Chromosome_36** |
| *IL13* | cg06967316 | 0.845 (0.020) | 0.802 (0.037) | 131993853 | TSS200 | 5 |
| *IL13* | cg07810967 | 0.935 (0.008) | 0.929 (0.011) | 131993925 | 1stExon | 5 |
| *IL13* | cg11798521 | 0.849 (0.017) | 0.826 (0.023) | 131996143 | 3'UTR | 5 |
| *IL13* | cg13566430 | 0.194 (0.021) | 0.183 (0.023) | 131992455 | TSS1500 | 5 |
| *IL13* | cg14523284 | 0.931 (0.007) | 0.927 (0.010) | 131993614 | TSS1500 | 5 |
| *IL13* | cg15329179 | 0.935 (0.010) | 0.93 (0.019) | 131993728 | TSS200 | 5 |
| *IL13* | cg24580593 | 0.943 (0.008) | 0.941 (0.009) | 11994061 | Body | 5 |
| *FLJ45983;GATA3* | cg01166071 | 0.046 (0.007) | 0.042 (0.008) | 8095687 | TSS1500 | 10 |
| *FLJ45983;GATA3* | cg04050331 | 0.020 (0.003) | 0.025 (0.004) | 8096088 | TSS1500 | 10 |
| *FLJ45983;GATA3* | cg04641787 | 0.043 (0.005) | 0.041 (0.006) | 8096154 | TSS1500 | 10 |
| *FLJ45983;GATA3* | cg04847548 | - | 0.045 (0.010) | 8096526 | TSS1500 |  |
| *FLJ45983;GATA3* | cg04982951 | 0.046 (0.006) | 0.044 (0.006) | 8096635 | TSS1500 | 10 |
| *FLJ45983;GATA3* | cg05071898 | 0.029 (0.008) | -- | 8095728 | TSS1500 | 10 |
| *FLJ45983;GATA3* | cg05671070 | 0.027 (0.005) | 0.029 (0.005) | 8095960 | TSS1500 | 10 |
| *FLJ45983;GATA3* | cg05721515 | -- | 0.067 (0.012) | 8095288 | Body;TSS1500 | 10 |
| *FLJ45983;GATA3* | cg06230736 | 0.038 (0.009) | 0.040 (0.009) | 8096650 | TSS1500;TSS200 | 10 |
| *FLJ45983;GATA3* | cg06870728 | -- | 0.076 (0.012) | 8095363 | Body;TSS1500 | 10 |
| Table A2. Information on the candidate CpG sites in the Th2 pathway. Mean and SD are calculated in beta values and for ages 10 and 18 years (continued). | | | | | | |
| **Gene** | **Name** | **Mean (SD) age10** | **Mean (SD) age18** | **MapInfo^§^** | **Location** | **Chromosome_36** |
| *FLJ45983;GATA3* | cg07516470 | -- | 0.031 (0.007) | 8095651 | TSS1500 | 10 |
| *FLJ45983;GATA3* | cg07578663 | 0.037 (0.004) | 0.033 (0.006) | 8096600 | TSS1500;TSS200 | 10 |
| *FLJ45983;GATA3* | cg07907745 | 0.123 (0.012) | 0.137 (0.019) | 8095493 | TSS200;TSS1500 | 10 |
| *FLJ45983;GATA3* | cg08347183 | 0.036 (0.007) | 0.032 (0.008) | 8096633 | TSS1500;TSS200 | 10 |
| *FLJ45983;GATA3* | cg08707112 | 0.058 (0.005) | 0.048 (0.005) | 8095764 | TSS1500 | 10 |
| *FLJ45983;GATA3* | cg09728012 | 0.025 (0.007) | 0.025 (0.005) | 8096305 | TSS1500 | 10 |
| *FLJ45983;GATA3* | cg11018337 | 0.026 (0.005) | 0.023 (0.01) | 8095495 | TSS200;TSS1500 | 10 |
| *FLJ45983;GATA3* | cg11731114 | 0.041 (0.011) | 0.039 (0.01) | 8096064 | TSS1500 | 10 |
| *FLJ45983;GATA3* | cg12730771 | 0.020 (0.004) | 0.021 (0.004) | 8095996 | TSS1500;TSS1500 | 10 |
| *FLJ45983;GATA3* | cg13431023 | 0.042 (0.006) | 0.042 (0.005) | 8096220 | TSS1500 | 10 |
| *FLJ45983;GATA3* | cg13543854 | 0.047 (0.007) | 0.042 (0.008) | 8095477 | TSS200;TSS1500 | 10 |
| *FLJ45983;GATA3* | cg13814485 | 0.026 (0.006) | 0.025 (0.008) | 8095500 | TSS200;TSS1500 | 10 |
| *FLJ45983;GATA3* | cg14098681 | 0.034 (0.007) | 0.031 (0.009) | 8096818 | TSS1500 ;1stExon | 10 |
| *FLJ45983;GATA3* | cg15187550 | 0.020 (0.006) | -- | 8096370 | TSS1500 | 10 |
| *FLJ45983;GATA3* | cg15330117 | 0.032 (0.006) | 0.034 (0.009) | 8096669 | TSS1500 ;1stExon | 10 |
| *FLJ45983;GATA3* | cg15852223 | 0.021 (0.003) | 0.017 (0.004) | 8096372 | TSS1500 | 10 |
| *FLJ45983;GATA3* | cg17566118 | 0.041 (0.007) | -- | 8095797 | TSS1500 | 10 |
| Table A2. Information on the candidate CpG sites in the Th2 pathway. Mean and SD are calculated in beta values and for ages 10 and 18 years (continued). | | | | | | |
| **Gene** | **Name** | **Mean (SD) age10** | **Mean (SD) age18** | **MapInfo^§^** | **Location** | **Chromosome_36** |
| *FLJ45983;GATA3* | cg17891011 | 0.030 (0.005) | 0.03 (0.005) | 8096152 | TSS1500 | 10 |
| *FLJ45983;GATA3* | cg18187680 | 0.038 (0.011) | 0.034 (0.006) | 8095825 | TSS1500 | 10 |
| *FLJ45983;GATA3* | cg18738647 | -- | 0.039 (0.006) | 8096158 | TSS1500 | 10 |
| *FLJ45983;GATA3* | cg19315863 | 0.021 (0.004) | 0.017 (0.004) | 8096597 | TSS1500;TSS200 | 10 |
| *FLJ45983;GATA3* | cg19679989 | 0.040 (0.006) | -- | 8096602 | TSS1500;TSS200 | 10 |
| *FLJ45983;GATA3* | cg19894747 | 0.033 (0.007) | -- | 8096386 | TSS1500 | 10 |
| *FLJ45983;GATA3* | cg20314737 | 0.056 (0.006) | 0.050 (0.006) | 8096579 | TSS1500;TSS200 | 10 |
| *FLJ45983;GATA3* | cg22647713 | 0.042 (0.007) | 0.043 (0.010) | 8095697 | TSS1500 | 10 |
| *FLJ45983;GATA3* | cg23058185 | 0.030 (0.005) | 0.031 (0.005) | 8095985 | TSS1500 | 10 |
| *FLJ45983;GATA3* | cg24647276 | 0.034 (0.006) | 0.029 (0.006) | 8096311 | TSS1500 | 10 |
| *FLJ45983;GATA3* | cg24797840 | 0.109 (0.019) | 0.103 (0.014) | 8095173 | Body;TSS1500 | 10 |
| *GATA3* | cg00463367 | 0.190 (0.023) | 0.168 (0.033) | 8103673 | Body | 10 |
| *GATA3* | cg01255894 | 0.064 (0.008) | 0.062 (0.007) | 8099218 | Body | 10 |
| *GATA3* | cg01522692 | 0.942 (0.011) | 0.947 (0.015) | 8117015 | 3'UTR | 10 |
| *GATA3* | cg03669298 | 0.064 (0.007) | 0.057 (0.009) | 8102210 | Body | 10 |
| *GATA3* | cg03935183 | 0.924 (0.013) | 0.925 (0.016) | 8100563 | Body | 10 |
| *GATA3* | cg04213746 | 0.955 (0.007) | 0.945 (0.009) | 8106003 | Body | 10 |
| Table A2. Information on the candidate CpG sites in the Th2 pathway. Mean and SD are calculated in beta values and for ages 10 and 18 years (continued) | | | | | | |
| **Gene** | **Name** | **Mean (SD) age10** | **Mean (SD) age18** | **MapInfo^§^** | **Location** | **Chromosome_36** |
| *GATA3* | cg04492228 | 0.185 (0.027) | 0.183 (0.036) | 8101513 | Body | 10 |
| *GATA3* | cg07989490 | 0.963 (0.006) | 0.958 (0.01) | 8117026 | 3'UTR | 10 |
| *GATA3* | cg10008757 | 0.073 (0.012) | 0.068 (0.009) | 8097183 | 5'UTR | 10 |
| *GATA3* | cg10089865 | 0.935 (0.011) | 0.932 (0.013) | 8100286 | Body | 10 |
| *GATA3* | cg10163955 | 0.731 (0.034) | 0.713 (0.042) | 8101402 | Body | 10 |
| *GATA3* | cg11430077 | 0.106 (0.021) | 0.095 (0.019) | 8099018 | Body | 10 |
| *GATA3* | cg11679455 | -- | 0.93 (0.012) | 8100761 | Body | 10 |
| *GATA3* | cg12181459 | 0.442 (0.061) | 0.325 (0.051) | 8098328 | Body | 10 |
| *GATA3* | cg12405139 | 0.929 (0.008) | 0.923 (0.011) | 8106035 | Body | 10 |
| *GATA3* | cg13409449 | 0.035 (0.005) | 0.036 (0.007) | 8097354 | 5'UTR | 10 |
| *GATA3* | cg14327531 | 0.062 (0.010) | 0.052 (0.009) | 8097331 | 5'UTR | 10 |
| *GATA3* | cg15043723 | 0.908 (0.009) | 0.884 (0.014) | 8106006 | Body | 10 |
| *GATA3* | cg15267232 | 0.070 (0.009) | 0.082 (0.013) | 8097689 | Body | 10 |
| *GATA3* | cg16267979 | -- | 0.901 (0.02) | 8100114 | Body | 10 |
| *GATA3* | cg17124583 | 0.049 (0.011) | 0.05 (0.019) | 8097641 | Body | 10 |
| *GATA3* | cg17489908 | 0.240 (0.030) | 0.225 (0.035) | 8101566 | Body | 10 |
| *GATA3* | cg18599069 | 0.060 (0.011) | 0.053 (0.007) | 8096991 | 5'UTR | 10 |
| Table A2. Information on the candidate CpG sites in the Th2 pathway. Mean and SD are calculated in beta values and for ages 10 and 18 years (continued) | | | | | | |
| **Gene** | **Name** | **Mean (SD) age10** | **Mean (SD) age18** | **MapInfo^§^** | **Location** | **Chromosome_36** |
| *GATA3* | cg19883813 | 0.045 (0.011) | 0.047 (0.01) | 8098005 | Body | 10 |
| *GATA3* | cg22770911 | 0.549 (0.036) | 0.484 (0.035) | 8101307 | Body | 10 |
| *GATA3* | cg22892607 | -- | 0.062 (0.01) | 8102583 | Body | 10 |
| *GATA3* | cg23687654 | 0.944 (0.009) | 0.94 (0.01) | 8117064 | 3'UTR | 10 |
| *GATA3* | cg25630514 | 0.658 (0.044) | 0.603 (0.078) | 8103415 | Body | 10 |
| *GATA3* | cg27409129 | 0.941 (0.007) | 0.936 (0.01) | 8111731 | Body | 10 |
| *STAT6* | cg00500522 | 0.030 (0.006) | 0.020 (0.007) | 57504387 | 5'UTR | 12 |
| *STAT6* | cg01063813 | 0.070 (0.020) | 0.054 (0.013) | 57504948 | 1stExon;5'UTR | 12 |
| *STAT6* | cg03848267 | 0.026 (0.004) | 0.026 (0.004) | 57506361 | TSS1500 | 12 |
| *STAT6* | cg07926491 | 0.943 (0.007) | 0.943 (0.009) | 57489971 | 3'UTR | 12 |
| *STAT6* | cg12693595 | 0.030 (0.004) | 0.031 (0.004) | 57504318 | 5'UTR | 12 |
| *STAT6* | cg14062067 | -- | 0.927 (0.011) | 57499258 | Body | 12 |
| *STAT6* | cg20779414 | 0.027 (0.005) | 0.026 (0.004) | 57505882 | TSS1500 | 12 |
| *STAT6* | cg25157914 | 0.035 (0.006) | 0.028 (0.004) | 57505529 | TSS1500 | 12 |
| *STAT6* | cg27320213 | 0.044 (0.004) | 0.038(0.004) | 57504988 | 1stExon;5'UTR | 12 |

**§** MapInfo refers to “genomic coordinates on the human genome” and it gives genomic location.

Figure A1. The means and standard deviation of CpG sites in genes IL4, IL4R, IL13, and STAT6 (data are in Table A2.)


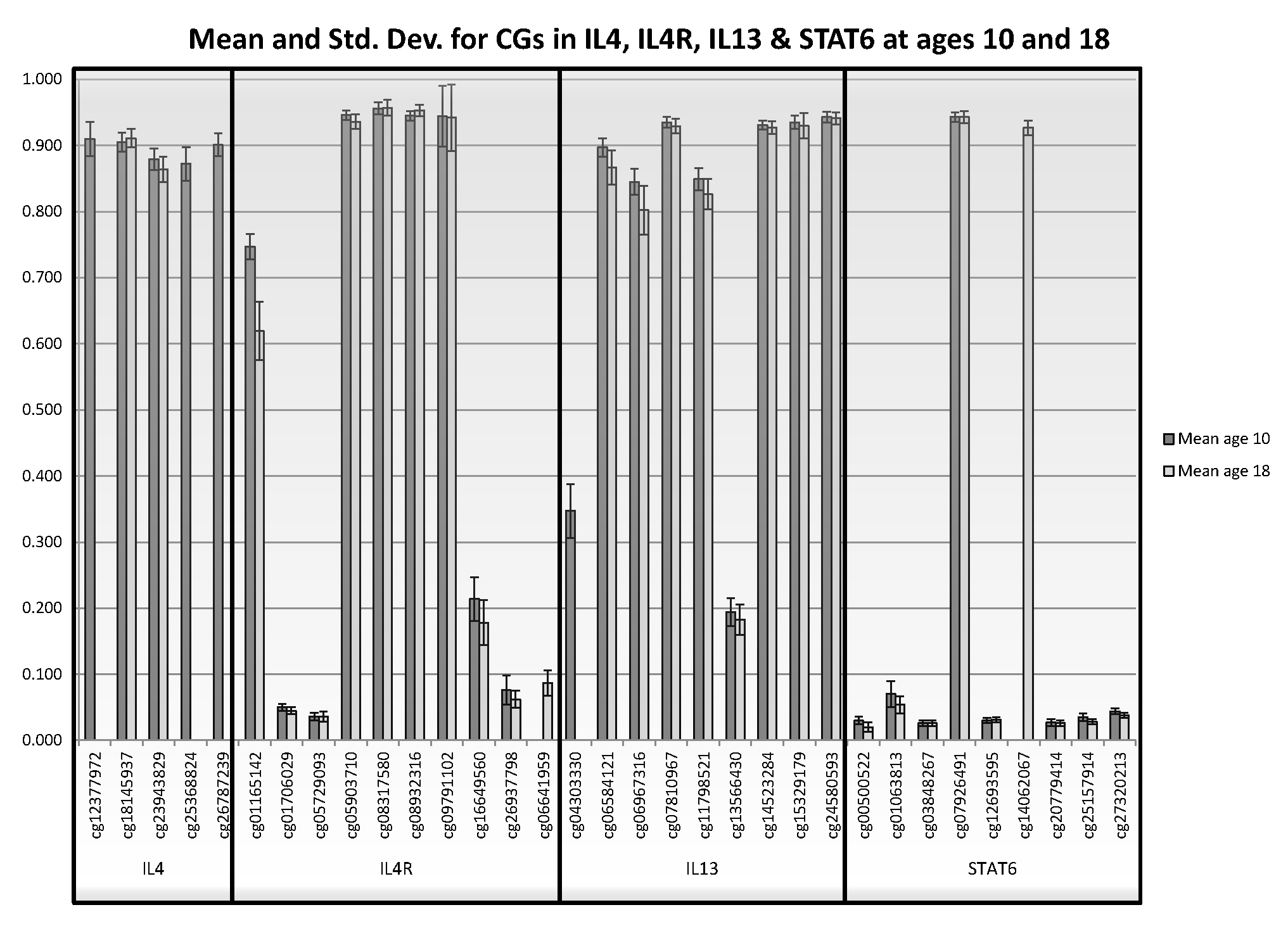


Figure A2. The means and standard deviation of CpG sites in the GATA3 gene (data are in Table A2.)


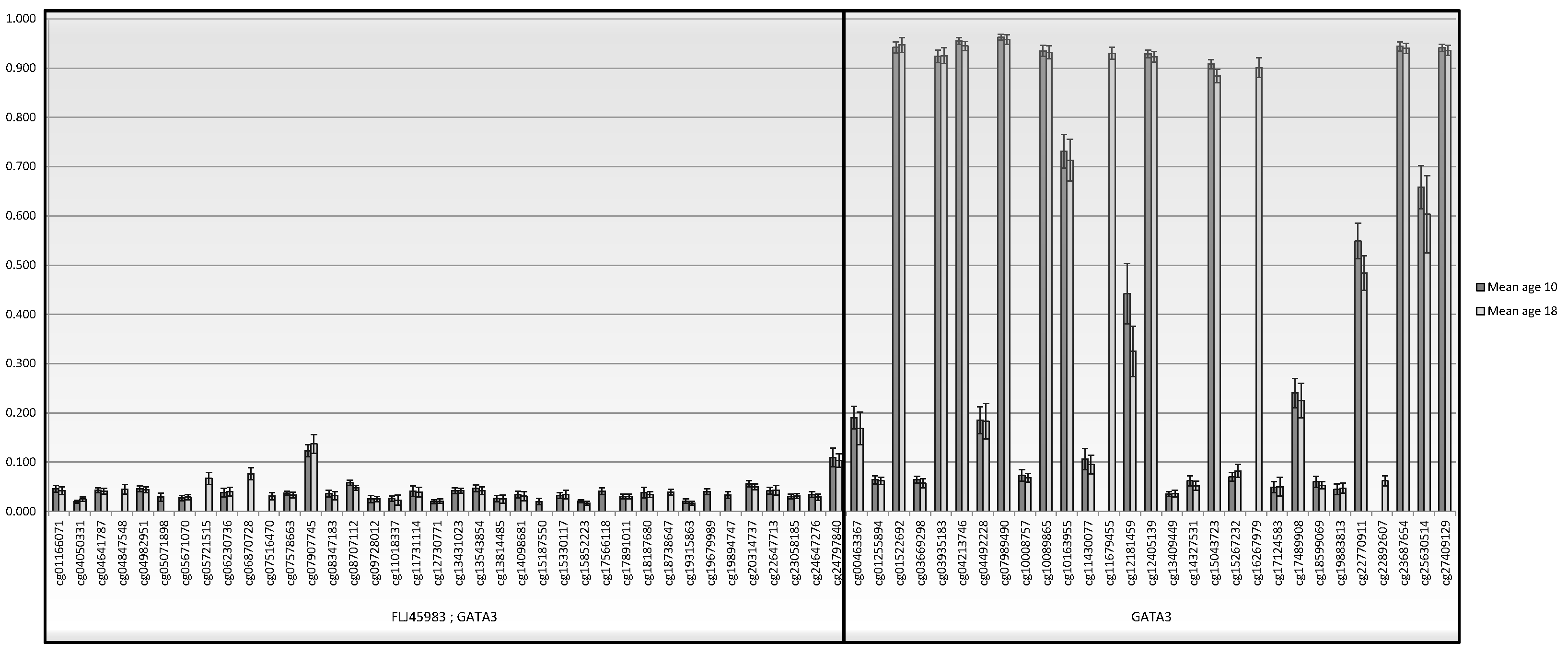

Supplement: Additional file 2: Table S2 — Information on the candidate CpG sites in the Th2 pathway. Mean and SD are calculated in beta values and for ages 10 and 18 years. Figure S1. The mean and SD of CpG sites in genes IL4, IL4R, IL13, and STAT6 (data are in Additional file 2: Table S2.). Figure S2. The mean and SD of CpG sites in the GATA3 gene (data are in Additional file 2: Table S2). [file 1868-7083-6-8-S2.docx]
